# Effect of co-occurring mutations in *TP53* gene and *TERT* promoter on the survival of bladder cancer patients

**DOI:** 10.3389/fimmu.2026.1771897

**Published:** 2026-03-09

**Authors:** Maria Lina Tornesello, Maria Carmela Piccirillo, Rosa Tambaro, Vittorio Simeon

**Affiliations:** 1 Molecular Biology and Viral Oncology Unit, Department of Translational Research, Istituto Nazionale Tumori IRCCS Fondazione G. Pascale, Napoli, Italy; 2 Clinical Trials Unit, Department of Translational Research, Istituto Nazionale Tumori IRCCS Fondazione G. Pascale, Napoli, Italy; 3 Uro-Gynaecological Clinical Experimental Medical Oncology, Department of Urology and Gynecology, Istituto Nazionale Tumori IRCCS Fondazione G. Pascale, Napoli, Italy; 4 Medical Statistics Unit, Mental, Physical Health and Preventive Medicine department, University of Campania 'Luigi Vanvitelli', Napoli, Italy

**Keywords:** bladder urothelial carcinoma, co-mutations, telomerase, TERT promoter mutations, TP53 mutations

## Abstract

**Background:**

Mutations in the *TP53* gene and telomerase reverse transcriptase promoter (*TERT*p) are among the most frequent genetic alterations in bladder cancer, but the clinical impact of their co-occurrence has not been fully explored. In this study, we assessed the mutational landscape as well as the prognostic significance of concurrent *TERT*p and *TP53* mutations in a cohort of bladder urothelial carcinoma patients.

**Methods:**

Using data from the cBioPortal database, we retrospectively analysed primary bladder urothelial carcinoma cases profiled with the Memorial Sloan Kettering-Integrated Mutation Profiling of Actionable Cancer Targets (MSK-IMPACT) assays. We investigated the relationships between tumour mutational burden (TMB), microsatellite instability (MSI), and somatic mutations. The Kaplan-Meier method was used to calculate patient overall survival. Log-rank testing and multivariable Cox proportional hazards modelling were used to evaluate prognostic factors.

**Results:**

Among the 1,111 cancer cases, 416 exhibited concurrent mutations in both *TERT*p and *TP53*, 387 harboured mutations exclusively in *TERT*p, 132 showed mutations only in the *TP53* gene, and 176 cases were double wild-type for both genetic regions (wt/wt). Overall survival was significantly longer in the wt/wt group compared to *TERT*p (HR 1.83, 95% CI 1.27 - 2.62, P<0.001), to *TP53* mutant alone (HR 1.84, 95% CI 1.19 - 2.85, P = 0.006) and to *TERT*p/*TP53* (HR 2.32, 95% CI 1.63 – 3.31, P<0.001) mutant groups. The presence of *TERT*p and *TP53* mutations was associated with higher tumour mutational burden (TMB ≥10 mutations/Mb) and increased microsatellite instability (MSI) scores (P < 0.001). The significant association between *TERT*p and *TP53* mutations was independently validated in a separate cohort.

**Conclusions:**

Bladder urothelial cancer can be stratified into biologically and clinically distinct subtypes on the basis of cancer driver mutations, with concomitant *TERT*p/*TP53* nucleotide changes strongly linked to reduced patients’ overall survival. These results suggest a potential cooperative interaction between mutant *TERT*p and *TP53* in the pathogenesis of bladder cancer, highlighting their significance as prognostic biomarkers and promising targets for novel therapeutic strategies.

## Introduction

Bladder cancer is one of the most common malignancies in the world, with approximately 613,791 cases and 220,349 deaths in 2022 (1). Several risk factors are involved in the development of bladder cancer, such as cigarette smoking, exposure to occupational and environmental contaminants, and advanced age (2, 3). Its incidence is about four times higher in men than in women, a disparity largely attributed to higher rates of smoking habit and occupational exposures among men, in addition to other biological and epidemiological factors (3–6).

Most bladder cancers are urothelial carcinomas, representing about 90% of cases in Western countries, with approximately 75% classified as non-muscle-invasive (NMIBC) and 25% as muscle-invasive (MIBC) or metastatic disease (7–9). The NMIBC are commonly low grade with favourable prognosis but with frequent recurrences, whereas MIBC are almost always high grade, aggressive, and often lethal (10, 11). A subset of NMIBC progresses to MIBC, underscoring the need for improved prediction of high-risk patients (12–16). A main characteristic of bladder urothelial cancer is the high frequency of multifocal synchronous and metachronous tumours (17). These lesions often share early trunk mutations and genomic aberrations, but also harbour distinct private alterations, reflecting both clonal relatedness and tumour heterogeneity (18–20).

Urothelial bladder cancer is among the most highly mutated cancers, with an average tumour mutation burden (TMB) of 7.7 per megabase and above 300 exonic mutations per tumour (21–23). The prevalent cancer driver mutations are localized in the telomerase reverse transcriptase (*TERT*) gene promoter (76%) and *TP53* (59%) followed by *RB1* (22%), *ERBB2* (14%), and *CDKN1A* (8.3%) (23–27).

Telomerase activity, generally undetectable in normal somatic cells, significantly increases in the majority of tumours causing elongation of telomeres at chromosomes ends and limitless replication of neoplastic cells (28). Multiple molecular mechanisms are involved in telomerase overexpression, including structural chromosomal alterations, copy number gains of the *TERT* locus, and activating mutations within the *TERT* promoter region (29). Recurrent activating mutations in *TERT*p, primarily hot spot mutations C228T and C250T, first identified in melanoma by Horn et al. (2013) and Huang et al. (2013), are among the most prevalent genetic alterations across various cancers, including bladder urothelial carcinoma (30–34). These mutations lead to the permanent activation of telomerase expression, by creating *de novo* consensus binding sites preferentially bound by the multimeric ETS factor GABPA/B1 (35–37). In bladder cancer, *TERT* expression from mutant (C228T) promoter alleles is selectively activated through binding of the transcription factor TRIM28, which undergoes mTORC1-mediated phosphorylation, leading to its dissociation from TRIM24 (38).

Mutations in the promoter region of the *TERT* gene (*TERT*p), besides being very frequent in urothelial carcinoma, represent early events in the transformation process since they are detected in the urinary exfoliated cells decades before the diagnosis of bladder cancer (34, 37, 39–41). In the Golestan cohort, which was monitored for up to 14 years, Hosen et al. found *TERT*p mutations in the urine of 47% of asymptomatic individuals who later developed primary bladder cancer, while no such mutations were detected in the matched control group that remained cancer-free (42). Accordingly, the identification of *TERT*p mutations in urinary exfoliated cells has emerged as a promising biomarker for early diagnosis of bladder cancer.

The tumour suppressor p53 is a central regulator of cellular homeostasis, controlling processes such as the cell cycle, DNA repair, apoptosis, autophagy, metabolism, and immune response (43). *TP53* is the most frequently mutated gene across human cancers, including bladder urothelial carcinoma, with the majority of alterations being non-synonymous mutations generating defective p53 proteins (44). These mutant forms typically lose tumour-suppressive activity, exert dominant-negative effects on wild-type p53, and in many cases acquire gain-of-function properties that promote tumour progression and oncogenesis (45). Urothelial bladder cancer patients with *TP53* inactivating mutations have poor overall survival but have a better response to immunotherapy due to increased tumour mutational burden and tumour associated antigens (46). Therefore, there is an urgent need for additional studies in bladder urothelial cancer clarifying how the type of *TP53* mutation within a tumour can best be used as a predictive biomarker.

The specific aims of this study were to provide a detailed genomic analysis of recurring mutations in bladder urothelial carcinoma and to evaluate the impact of co-occurring mutations in *TERT*p and in *TP53* gene on patient’s clinical outcome in order to possibly develop a bladder cancer informed molecular classification system.

## Methods

### Patients and data collection

The cBioPortal database was queried to identify bladder cancer datasets, resulting in 21 independent genomic studies publicly available for analyses (**Supplementary Table S1**). Studies were categorized according to the availability of *TERT*p mutation data, along with annotated somatic mutations in coding regions, and survival time. *TERT*p mutation status along with survival information, were available only for the Bladder Cancer (MSK, Cell Reports 2022) study, which was therefore included as the principal dataset in the analyses (47). The Bladder Urothelial Carcinoma SWOG S1314 Trial (MSK, JCO Precis Oncol 2024) cohort containing information regarding *TERT*p but not survival time was analysed as independent validation set to confirm the robustness of association between *TP53* and *TERT*p (48).

The principal dataset included a total of 1,659 samples from 1,244 bladder cancer patients treated and enrolled at the Memorial Sloan Kettering Cancer Center (MSK) from 1999 to 2021 (ClinicalTrials.gov, NCT01775072) (47). All samples have been analysed with the MSK-IMPACT^®^ (Integrated Mutation Profiling of Actionable Cancer Targets), targeted sequencing assays developed at Memorial Sloan Kettering Cancer Center that detect clinically relevant genetic alterations and enable mutation profiling across a broad range of genes. Tumours were profiled using the MSK-IMPACT341 (n = 46), MSK-IMPACT410 (n = 196), MSK-IMPACT468 (n = 825), and MSK-IMPACT505 (n = 44) panels, all of which included the genes selected for this study.

Applying the selection criteria “*cancer type = bladder cancer*” and “*cancer type detailed = bladder urothelial carcinoma*” yielded a cohort of 1,111 patients whose samples derived from primary tumour (n=960), local recurrence (n=1) and metastasis (n=150). Demographic, clinical, pathologic and treatment information, along with the somatic mutation types and mutation sites, were retrieved through the search options in cBioPortal and downloaded as.tsv files. Patients’ information included sex, age at bladder cancer diagnosis, smoking habit (active, former, never), treatment history, survival time (months). Tumour features included the histological classification, TMB, microsatellite instability (MSI) score and prevalent gene mutations (47). The primary objective of the study was to identify differences in the overall survival between four groups of patients according to *TERT*p and *TP53* mutation status: *TERT*p and *TP53* wild type tumours (wt/wt), *TERT*p mutant and *TP53* wild type tumours (*TERT*p), *TERT*p wild type and *TP53* mutant tumours, *TERT*p and *TP53* double mutant tumours (*TERT*p/*TP53*).

The *Bladder Urothelial Carcinoma SWOG S1314 Trial (MSK, JCO Precis Oncol 2024)* cohort including 184 cases of bladder urothelial carcinoma that were analysed with the MSK-IMPACT assay, was used to validate the significance of *TERT*p and *TP53* co-occurrence (**Supplementary Figure S2**) (48).

### Mutation annotation and co-mutations definition

Complete datasets of genes harbouring somatic mutations with a frequency ≥10% were queried using the search function in cBioPortal, and the corresponding files were downloaded for further analysis. For *TERT*p, only tumour cases harbouring recurrent mutations at positions C228T and C250T, which have been shown to activate *TERT* expression, were classified as *TERT*p mutant, all other cases were assigned to the *TERT*p wild-type group. In addition, we assessed the co-occurrence of cancer driver mutations in *TERT*p and *TP53* genes as well as their impact on the overall survival of bladder cancer patients. Other frequently mutated genes included *KDM6A*, *ARID1A*, *KMT2D*, *FGFR3*, *PIK3CA*, *RB1*, *ELF3*, *KMT2C*, *CREBBP*, *ERBB2*, *CDKN1A*, *ATM*, *FAT1*, *STAG2*, *KMT2A*, *EP300*, *TSC1*, *ERBB3* and *ERCC2*, and their relationship with *TERT*p and *TP53* mutations was evaluated. Co-mutations were classified at the gene level: two genes were considered to be co-mutated if they each had at least one mutation, the actual number of mutations in each gene were not taken into consideration. For example, if one sample harboured two mutations in gene A and three mutations in gene B, only one co-mutation was considered. Each gene was assigned a binary value 0 or 1 for wild type and mutated status, respectively.

### Statistical analysis

Categorical variables were expressed as numbers and percentages, continuous variables as medians and interquartile ranges (IQRs). The presence of missing data has been reported in **Table 1**. Differences between groups organized by tumour mutation status (wt/wt, *TERT*p, *TP53*, and *TERT*p/*TP53*) and clinical covariates were assessed using the Kruskal–Wallis test or the Fisher exact test. Co-mutations analyses were performed using the Chi-square test and the results were adjusted for the false discovery rate with q-value <0.05 considered as statistically significant. The mutations in *TP53* gene and *TERT*p as well as in other cancer driver genes and their association with survival probability in bladder cancer patients have been evaluated. The survival time was calculated from the date of diagnosis to the date of death from bladder cancer or the last follow-up visit. A log-rank test was performed to identify the significance of differences between the survival curves. Univariate analyses or multivariate Cox regression models were used to assess the hazard ratio (HR) with 95% confidence intervals (CI) to assess the prognostic effect of *TERT*p and/or *TP53* status, age at diagnosis, smoking history, MSI score and TMB. Multivariable Cox regression analyses were performed using a complete-case approach, including only patients with full documentation for all variables entered the model. The proportional hazards assumption for the Cox models was formally evaluated and confirmed by evaluating the independence between Schoenfeld residuals and time. P values <0.05 were considered statistically significant. All analyses were performed using RStudio (R version 4.3.2) with R packages “tidyverse”, “janitor”, “dplyr”, “gtsummary”, “table1”, “survival” and “survminer”.

**Table 1:** Patient clinical characteristics.

Variables	Total (N = 1,111)
Age	
Median (IQR)	66 (58 - 73)
Missing	106 (9.5%)
Sex (%)	
Female	261 (23%)
Male	849 (76%)
Missing	1 (0.1%)
Smoking (%)	
Never	363 (33%)
Active, former	654 (59%)
Missing	94 (8%)
Tumour grade (%)	
G1-2	247 (22%)
G3	796 (72%)
Missing	68 (6.1%)
Intravesical treatment	
Naive	748 (67%)
Treated	266 (24%)
Missing	97 (8.7%)
Systemic treatment	
Naive	828 (75%)
Treated	172 (15%)
Missing	111 (10%)
MSI status (%)	
Instable H	9 (1%)
Instable L	16 (1%)
Stable	1082 (97%)
Missing	4 (0.4%)
MSI score	
Median (IQR)	0.2 (0 - 0.66)
Missing	4 (0.4%)
TMB (mutations/Mb)	
Median (IQR)	9.5 (5.9 - 17)
N. Mutations per sample	
Median (IQR)	11 (7.0 - 18)
Missing	24 (3%)

## Results

### Patients’ characteristics

We performed a retrospective study of 1,111 bladder urothelial cancer cases. All demographic, pathologic and genomic data were collected for patients with localized or metastatic bladder urothelial carcinoma referring to Memorial Sloan Kettering Cancer Center (MSK) from 1999 to 2021, and publicly available through the https://www.cbioportal.org/. Molecular data were available for 960 primary tumours, 150 metastasis and one local recurrence samples. The clinical characteristics of the patients are reported in **Table 1**. The median age at cancer diagnosis was 66 years (IQR range, 58–73 years). The patient group included 261 females and 849 males (sex ratio 1:3). The majority of patients were former smokers (n=538), followed by never smokers (n=363), and active smokers (n=116). Overall, 64% of the patients were active or former smokers and 36% never smoked. Most bladder urothelial carcinomas were poorly differentiated grade 3 (G3, 72%). A group of patients (24%) received intravesical therapy, including BCG, chemotherapy or both. A second group received systemic therapy (15%), including chemotherapy, immunotherapy or both therapies. Patients who received intravesical or systemic treatments were grouped in “intravesical treated” and in “systemic treated”, respectively, without further distinction by type of therapy. A total of 748 patients were naïve to any treatment.

### Gene mutations in bladder cancer

The median MSI score was 0.2 (IQR, 0 - 0.66), with most tumours classified as MS stable (97%) having a score below 3.5 (**Table 1**). Few cases (1%) had low MS instability (MS Instable L, score equal or higher than 3.5 and lower than 10) or (1%) high MS instability (MS Instable H, score equal or higher than 10). The median TMB score for the 1,111 patients was 9.5 (IQR, 5.9 – 17). Genetic alterations, including gene amplification and copy number variations, were rare, occurring in less than 5% of cases at both the *TERT* locus and *TP53* (**Supplementary Figure 1**). The most frequent mutations were single nucleotide changes in *TERT*p, with the hotspot mutations C228T and C250T observed in 62.3% (692/1,111) and 10% (111/1,111) of cases, respectively. Mutations other than C228T and C250T, whose impact on *TERT* expression has not been characterized, accounted for 5.2% (58/1,111). In the analyses, only tumours harbouring C228T or C250T mutations, which are known to activate *TERT* expression, were classified as *TERT*p mutant, while all remaining tumours were assigned to the wild-type group. Among genes with mutation rates above 10%, eleven are classified as oncosuppressors (*TP53*, *KDM6A*, *ARID1A*, *CREPPB*, *KMT2C*, *RB1*, *CDKN1A*, *FAT1*, *ATM*, *ELF3*, *TSC1*) and nine as oncogenes (*FGFR3*, *KMT2D*, *PIK3CA*, *ERBB2*, *ERCC2*, *STAG2*, *EP300*, *KMT2A* and *ERBB3*), according to https://bioinfo.uth.edu/TSGene/. The *TP53* mutations were the most frequent, occurring in 49% of cases (548/1,111). In addition, a high mutation rate was observed in genes involved in chromatin remodelling, such as histone methyl transferases *KMT2D*, *KMT2C* and *KMT2A*, the histone demethylase *KDM6A*, histone acetyltransferases *EP300* and *CREBBP*, as well as *ARID1A*, which is a part of chromatin SWI/SNF nucleosome-remodelling complex.

We compared the mutation frequencies of mutant genes between *TERT*p wild type and *TERT*p-mutant bladder cancer samples (**Supplementary Table S2**). Overall, nine genes exhibited significantly higher mutation frequencies, after multiple testing correction (q < 0.05), in *TERT*p-mutant compared with wild-type bladder cancer cases. Such genes included *CDKN1A, RB1, ERBB2, ARID1A, PIK3CA, TSC1, TP53, ELF3, KMT2A*. Functionally, these co-mutations involve both tumour suppressors (e.g., *CDKN1A, RB1, ARID1A, TSC1, TP53*, *ELF3*) and oncogenes (e.g., *ERBB2*, *PIK3CA, KMT2A)*, suggesting that *TERT*p mutations often coexist with other driver alterations that promote tumour proliferation, genomic instability, and telomerase activation. Notably, these mutant genes are functionally involved in cell cycle regulation (*CDKN1A*, *RB1*, *TP53*), chromatin remodelling (*ARID1A*, *KMT2A*), and activation of oncogenic signalling pathways (*ERBB2*, *PIK3CA*).

Moreover, distinct mutational patterns were observed between *TP53* mutant and *TP53* wild type bladder cancers (**Supplementary Table S3**). Four genes showed significant higher mutation frequencies in *TP53*-mutant cases (q<0.05), including *RB1*, *ERBB2*, *TERT*p, *TSC1*, suggesting cooperative effect in tumour progression (**Supplementary Table S3**). Of note, in *TP53*-wild-type tumours the most frequent mutations (q<0.05) were identified in *FGFR3*, *CDKN1A*, and *STAG2*, while typically absent in *TP53*-mutant cases, indicating that alterations in these genes and *TP53* are mostly mutually exclusive.

The *TERT*p/*TP53* double-mutant subgroup displayed high frequency of driver mutations in several genes, such as *RB1, ERBB2, ARID1A* and *KMT2A*, with *ERBB2* exhibiting the most marked shift (13% *versus* 24%), (**Table 2**). Among tumour suppressors, *RB1* displays a striking increase in mutations (6.8% *versus* 36%), underscoring extensive genomic instability associated with *TERT*p/*TP53* co-mutations. Conversely, other tumour suppressor genes, such as *FGFR3* (33% in wild type versus 12% in mutants) are significantly more mutated in the wild-type group. This suggests the existence of distinct oncogenic pathways in tumours that retain wild type *TERT*p/*TP53*, characterized by an alternative mutation spectrum, particularly involving *FGFR3*. Other genes, such as *ARID1A, KMT2A* and PIK3CA also show statistically significant differences, though with more moderate effect sizes. After false-discovery-rate correction, these associations remain robust (q < 0.05 for all significant genes), reinforcing their biological relevance.

**Table 2:** Distribution of mutant genes in *TERT*p and *TP53* wild type versus *TERT*p and *TP53* mutant cancer cases.

Gene name	Gene type	*TERT*p/*TP53* Wild type N = 176 (%)	*TERT*p/*TP53* Mutant N = 441	p-value*^1^*	q-value*^2^*
*FGFR3*	Oncosuppressor	58 (33%)	52 (12%)	<0.001	**<0.001**
*RB1*	Oncosuppressor	12 (6.8%)	158 (36%)	<0.001	**<0.001**
*ERBB2*	Oncogene	22 (13%)	107 (24%)	0.001	**0.005**
*ARID1A*	Oncosuppressor	41 (23%)	151 (34%)	0.008	**0.028**
*KMT2A*	Oncogene	13 (7.4%)	65 (15%)	0.013	**0.039**
*PIK3CA*	Oncogene	22 (13%)	88 (20%)	0.029	0.076
*STAG2*	Oncogene	28 (16%)	46 (10%)	0.059	0.14
*CDKN1A*	Oncosuppressor	12 (6.8%)	50 (11%)	0.092	0.2
*ERBB3*	Oncogene	15 (8.5%)	58 (13%)	0.11	0.2
*ATM*	Oncosuppressor	30 (17%)	58 (13%)	0.2	0.4
*EP300*	Oncogene	18 (10%)	61 (14%)	0.2	0.4
*TSC1*	Oncosuppressor	12 (6.8%)	43 (9.8%)	0.2	0.4
*ERCC2*	Oncogene	21 (12%)	66 (15%)	0.3	0.5
*KDM6A*	Oncosuppressor	60 (34%)	134 (30%)	0.4	0.5
*CREBBP*	Oncosuppressor	21 (12%)	59 (13%)	0.6	0.8
*KMT2D*	Oncogene	46 (26%)	119 (27%)	0.8	0.9
*ELF3*	Oncosuppressor	17 (9.7%)	46 (10%)	0.8	0.9
*KMT2C*	Oncosuppressor	29 (16%)	69 (16%)	0.8	0.9
*FAT1*	Oncosuppressor	22 (13%)	54 (12%)	>0.9	>0.9

*^1^*Pearson’s Chi-squared test.

*^2^*False discovery rate correction for multiple testing. Significant p-values are indicated in bold.

Mutation analysis in the validation cohort, which included 184 bladder urothelial carcinoma cases, confirmed that *TERT*p and *TP53* mutations were the most frequent genetic alterations, occurring in 80.4% (148/184) and 65.2% (120/184) of cases, respectively. A significant co-occurrence between *TERT*p and *TP53* mutations was also observed (**Supplementary Figure S1**).

### *TERT*p and *TP53* mutations in urothelial bladder cancer and overall survival analyses

Among the 1,111 urothelial bladder cancer patients, 176 had tumours with wild-type *TERT*p and *TP53* (wt/wt), 387 had *TERT*p-mutated tumours (*TERT*p mut/*TP53* wt), 132 had *TP53*-mutated tumours (*TP53* mut/*TERT*p wt), and 416 were *TERT*p and *TP53* double mutant tumours (*TERT*p mut/*TP53* mut) (**Table 3**). There were no significant differences between groups in the median age (P = 0.3), sex (P = 0.8), or smoking history (P = 0.5). The percentage of tumours classified as grade G3 was higher in the *TERT*p/*TP53* co-mutated tumours (92%) than in other groups (68% of *TERT*p mut/*TP53* wt and 82% of TERTp wt/TP53 mut tumours compared to 54% of wt/wt, P<0.001). Furthermore, patients with double mutant tumours had higher mutation burden (13 mutations/Mb) versus the other groups (8 mutations/Mb for wt/wt, 10 for TERTp wt/TP53 mut wt, and 8 for TERTp wt/TP53 mut, P< 0.001).

**Table 3:** Baseline patients and tumour characteristics by *TERT*p, *TP53* and *TERT*p/*TP53* mutation status.

Characteristic	N	wt/wt, N = 176	*TERT*p mut/*TP53* wt N = 387^1^	TERTp wt/*TP53* mut N = 132^1^	*TERT*p mut/*TP53* mut N = 416^1^	p-value^2^
Median age, y	1,004	65 (18–87)	66 (30–91)	66 (31–92)	67 (34–89)	0.3
Sex	1,110					0.8
Female		44 (25%)	90 (23%)	34 (26%)	93 (22%)	
Male		131 (75%)	297 (77%)	98 (74%)	323 (78%)	
Smoking history	1,017					0.5
Never		66 (41%)	124 (34%)	44 (35%)	129 (35%)	
Former/active		96 (59%)	236 (66%)	82 (65%)	240 (65%)	
Tumour grade	1,043					**<0.001**
G1-2		74 (46%)	120 (32%)	24 (18%)	29 (8%)	
G3		87 (54%)	253 (68%)	107 (82%)	349 (92%)	
MSI type	1,105					**0.023**
Instable H		5 (2.9%)	1 (0.3%)	2 (1.5%)	12 (0.2%)	
Instable L		1 (0.6%)	6 (1.6%)	3 (2.3%)	6 (1.4%)	
Stable		168 (97%)	378 (98%)	127 (96%)	409 (98%)	
MSI score	1,107	0.09 (0, 0.40)	0.17 (0, 0.56)	0.26 (0, 0.93)	0.27 (0.05, 0.83)	**<0.001**
TMB (n. mut/Mb)	1,087	8 (4, 14)	10 (6, 16)	8 (5, 13)	13 (9, 21)	**<0.001**

^1^Median (Minimum–Maximum); n (%); Median (IQR).

^2^Kruskal-Wallis rank sum test; Pearson’s Chi-squared test. Significant p-values are indicated in bold.

Univariate Cox regression analysis showed that several clinical and molecular factors were associated with overall survival (**Table 4**). In the entire cohort, older age, smoking history, higher tumour grade (G3), and the presence of *TERT*p or *TP53* mutations, individually or combined, were significantly associated with poorer survival. These associations were primarily observed in the untreated patients subgroup, where age, smoking, high-grade tumours, and *TERT*p and *TP53* alterations remained strong predictors of unfavourable outcomes. In contrast, among patients with systemic treatments, no variables were significantly associated with survival, suggesting that therapy may attenuate their negative prognostic effects. Interestingly, co-occurring mutations in *TERT*p and *TP53* were linked to improved survival in systemic treated patients, although this did not reach statistical significance (HR = 0.77, 95% CI: 0.35–1.66, p = 0.5), highlighting a potential predictive effect on treatment response.

**Table 4:** Univariate Cox-proportional hazard model for evaluating overall survival in all patients, untreated and systemic treated patients.

Variables	All patients			Untreated patients				Treated patients^**^		
	HR	95%CI	P value^*^		HR	95%CI	P value^*^	HR	95%CI	P value^*^
Age	1.02	1.01 - 1.03	**<0.001**		1.04	1.02 - 1.05	**<0.001**	0.98	0.97 - 1.00	0.11
Sex male	1.05	0.83 - 1.34	0.7		1.05	0.74 - 1.50	0.8	0.88	0.52 - 1.49	0.6
Smoking^***^	1.60	1.26- 2.01	**<0.001**		1.69	1.20- 2.38	**0.003**	1.36	0.86 – 2.14	0.2
Grade G3	3.55	2.56-4.93	**<0.001**		3.41	2.15 – 5.41	**<0.001**	1.64	0.72 – 3.75	0.2
MSI score	0.96	0.90- 1.02	0.2		0.95	0.86 - 1.06	0.4	0.94	0.85 - 1.04	0.2
TMB	0.99	0.99 - 1.00	0.1		1.00	0.99 - 1.01	0.7	0.99	0.97 - 1.00	0.12
*TERT*p mut/*TP53* wt^****^	1.83	1.27 - 2.62	**0.001**		2.17	1.20 – 3.90	**0.010**	0.86	0.40 - 1.85	0.7
TERTpwt/*TP53* mut^****^	1.84	1.19 – 2.85	**0.006**		2.69	1.38 – 5.23	**0.004**	0.97	0.41 - 2.32	>0.9
*TERT*p mut/*TP53* mut^****^	2.32	1.63 – 3.31	**<0.001**		3.21	1.81 – 5.70	**<0.001**	0.77	0.35 – 1.66	0.5

^*^Log rank test. Significant p-values are indicated in bold.

^**^Treated patients those who received systemic treatment (chemotherapy or immunotherapy).

^***^Smoking includes active and former smokers.

^****^Mutations and co-mutations versus wt/wt.

A multivariate Cox proportional hazards regression analysis was performed to assess the independent prognostic impact of clinical and molecular variables on overall survival in untreated patients (**Table 5**). Increasing age (HR = 1.03, 95% CI 1.02–1.05, p < 0.001), smoking history, including both current and former smokers (HR = 1.56, 95% CI 1.10–2.21, p = 0.012), higher tumour grade (G3, HR = 2.97, 95% CI 1.83–4.82, p < 0.001) were significantly associated with higher mortality risk. With respect to pathogenic variants, *TERT*p mutations (*TERT*p mut/*TP53* wt) were independently associated with increased risk of death (HR = 1.88, 95% CI 1.05–3.40, p = 0.035). *TERT*p mutations (*TERT*p wt/*TP53* mut) showed a borderline association with poorer survival (HR = 1.95, 95% CI 1.00–3.83, p = 0.051). Notably, the presence of concurrent TERTp and TP53 mutations (*TERT*p mut/*TP53* mut) conferred the highest risk and remained a significant independent predictor of poor outcome (HR = 2.06, 95% CI 1.15–3.70, p = 0.015), suggesting an additive or synergistic negative prognostic effect in untreated patients.

**Table 5:** Multivariable Cox proportional hazards model assessing the independent prognostic value of TERTp, TP53, and TERTp/TP53 co-mutations, adjusting for age, smoking history, and tumour grade, in bladder cancer patients who did not receive intravesical or systemic therapy.

Variables	Multivariate - Untreated patients		
	HR	95%CI	P value***
Age	1.03	1.02- 1.05	**<0.001**
Smoking^*^	1.56	1.10 - 2.21	**0.012**
Tumour grade G3	2.97	1.83 -4.82	**<0.001**
*TERT*p mut/*TP53* wt^**^	1.88	1.05 -3.40	**0.035**
*TERT*p wt*/TP53* mut^**^	1.95	1.0 – 3.83	0.051
*TERT*p mut/*TP53* mut^**^	2.06	1.15 – 3.70	**0.015**

^*^Smoking includes active and former smokers.

^**^Mutations and co-mutations versus wt/wt. ***Significant p-values are indicated in bold.

The Schoenfeld residual-based test of the proportional-hazards assumption for overall survival is reported in **Supplementary Table S4**. For all variables (age, smoking status, and mutant groups), p-values are > 0.05 and rho values are small, indicating no evidence of violation of the proportional-hazards assumption and supporting the validity of the Cox model.

**Figure 1:**
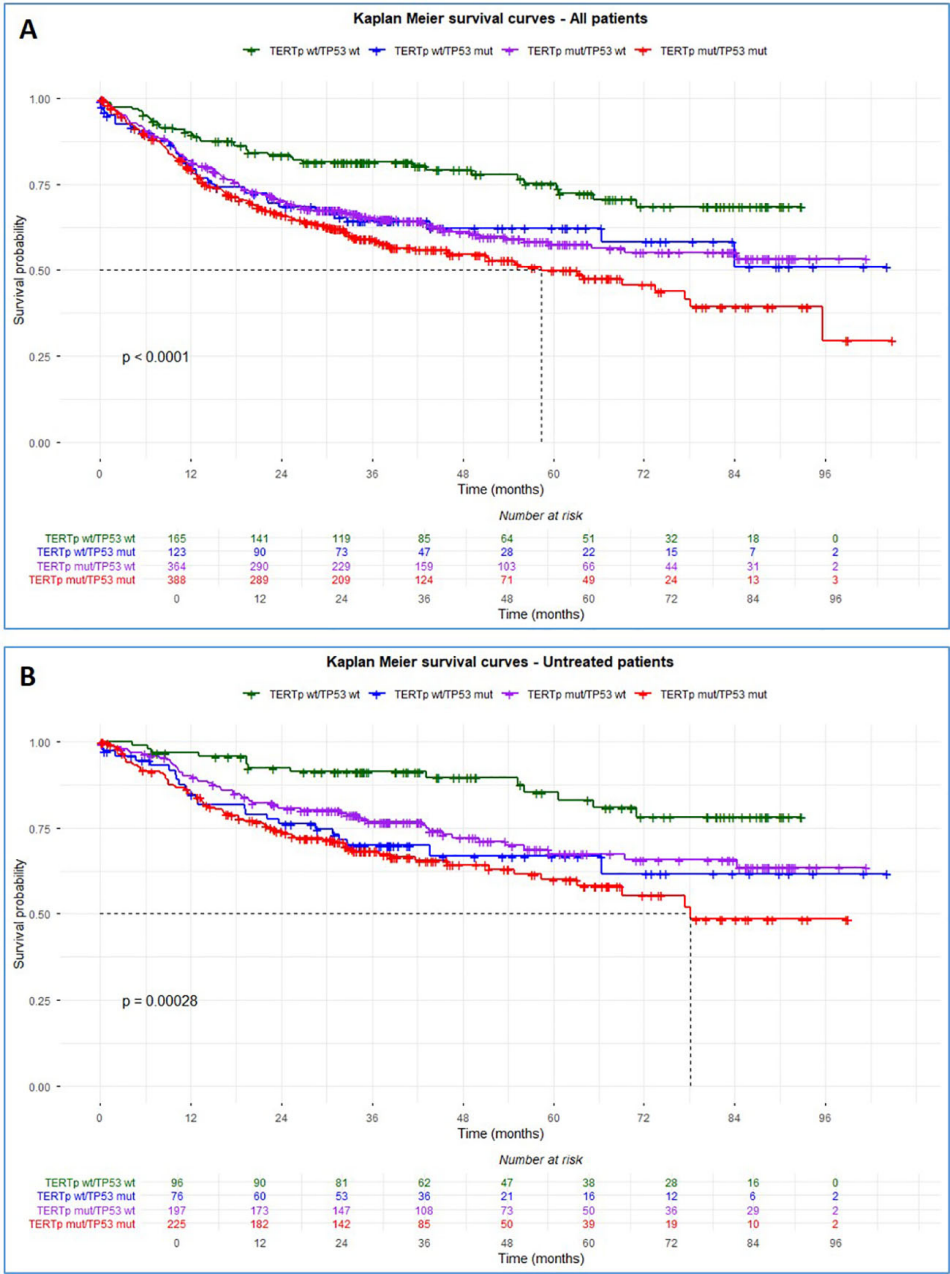
Kaplan-Meier survival analysis according to *TERT*p and *TP53* mutation status. **(A)** Kaplan-Meier survival curves for all patients stratified by combined *TERT*p and *TP53* mutation status. **(B)** Kaplan-Meier survival curves for untreated patients only.

To investigate the prognostic impact of concurrent *TERT*p and *TP53* mutations, we performed Kaplan–Meier survival analyses stratifying patients according to their mutational status. As shown in **Figure 1**, in the analysis including all patients, survival outcomes differed significantly among the four groups (wt/wt, *TERT*p wt/*TP53* mut, *TERT*p mut/*TP53* wt, and *TERT*p mut/*TP53* mut; log-rank p <0.001), (**Figure 1A**). Patients harbouring concurrent *TERT*p and *TP53* mutations exhibited the poorest survival, with a marked decline in survival probability beginning around 24–30 months. Whereas patients with *TERT*p wt/*TP53* wt demonstrate the most favourable prognosis, with the median not reached within the observed follow-up. Single-mutant subgroups (*TERT*p mut/*TP53* wt and *TERT*p wt/*TP53* mut) showed intermediate outcomes. To exclude potential confounding effects of therapy, we next examined survival in the subset of untreated patients. The same pattern was observed, with significantly worse outcomes for the *TERT*p mut/*TP53* mut group (log-rank p < 0.001), (**Figure 1B**). Additionally, Kaplan–Meier analyses in the untreated cohort demonstrated that older age (≥65 years), former or active smoking, and high-grade (G3) tumours were associated with lower overall survival probability (log-rank p = 0.0088, 0.0024, and <0.0001, **Supplementary Figure S3**). Median survival was not reached in any group, consistent with the observation that co-occurring mutations in *TERT*p and *TP53* reduced survival probability.

In patients who received systemic treatment (**Supplementary Figure S2**), Kaplan–Meier survival analysis stratified by *TERT*p and *TP53* mutations showed no significant differences in overall survival among the four groups (log rank p = 0.84). Survival curves largely overlapped and median survival times were comparable across groups. Conversely, among patients treated with intravesical therapy, overall survival differed significantly according to *TERT*p and *TP53* mutational status (log rank p = 0.029). Patients harbouring concurrent *TERT*p and *TP53* mutations exhibited the earliest decline in survival and reached median survival earlier than other groups. Patients with either *TERT*p or *TP53* mutations alone showed intermediate outcomes, whereas patients with wild-type *TERT*p and *TP53* demonstrated the most favourable survival.

## Discussion

For many years, studies on cancer molecular profiling have primarily been based on whole-exome sequencing, which largely excludes non-coding regions (49). Consequently, nucleotide alterations in regulatory elements such as *TERT*p C228T and C250T mutations and their coexistence with other cancer driver genes have not been systematically investigated (50). More recently, the use of whole-genome sequencing and targeted sequencing panels such as MSK-IMPACT has enabled the inclusion of these non-coding regions, allowing for the detection of *TERT*p mutations together with other clinically relevant genetic alterations (32, 41).

In the present study, we assessed the presence of concomitant *TERT*p mutations in bladder urothelial cancer, and we observed a cooperative association between co-occurring mutations in *TERT*p and *TP53*, which are shown to influence the clinical outcome. The results showed that 72% and 49% of urothelial bladder carcinoma harboured mutations in *TERT*p (C228T and C250T) and in *TP53* gene, respectively. Notably, 37% of tumours carried concurrent *TERT*p and *TP53* mutations, reflecting a statistically significant tendency toward co-mutation. The significant co-occurrence of TERT promoter and TP53 mutations was validated in the Bladder Urothelial Carcinoma SWOG S1314 trial cohort, confirming the robustness of this association (48). Nevertheless, the molecular pathways connecting these alterations and their potential cooperative effects remain to be determined. Mutational profiles of *TERT*p and *TP53* did not differ significantly by sex, age, or smoking history. However, the marked sex imbalance (849 males versus 261 females) is consistent with epidemiological data that bladder cancer incidence is approximately four times higher in men than in women (51).

Emerging evidence suggests that sex disparity may arise from biological differences in how oncogenic mutations are subjected to selection and accumulation in the bladder urothelium (52). A recent study demonstrated that the type of genetic alterations identified in mutant clones of normal urothelium differs markedly by sex, age and smoking history (53). Mutational analyses, performed by ultradeep sequencing across 16 genes, have shown that normal urothelium in males exhibits a significant higher burden of truncating mutations in *RBM10*, *CDKN1A*, and *ARID1A* genes compared to females. This sex-specific clonal selection may contribute to the higher incidence of bladder cancer in men by expanding premalignant fields long before tumour development. *TERT*p mutations have also been shown to drive clonal cell expansions in normal urothelium in both males and females, but they are detected almost exclusively in individuals over 55 years of age and are more prevalent among those with a history of smoking (53).

Mutations in TERTp showed significant enrichment of mutations in oncosuppressors (*ARID1A*, *CDKN1A*, *RB1*, *TP53*) and in oncogenes (*ERBB2*, *PIK3CA*, *KMT2A*), indicating that telomerase activation frequently co-occurs with additional genetic events that enhance proliferative capacity and genomic instability. Conversely, cancers harbouring *TP53* mutations showed higher frequencies of *RB1* nucleotide changes, whereas *TP53* wild-type tumours were enriched for alterations in *FGFR3*, *ELF3*, *CDKN1A*, and *STAG2*. Tumours carrying *TERT*p/*TP53* mutations were characterized by a sharp increase in *RB1* mutations (6.8% to 36%). On the other end, *TERT*p/*TP53* wild-type tumours carry a significant high number of mutations in *FGFR3* gene (33%).

Similar patterns of co-mutations were observed in the AACR Project GENIE, with *TP53* mutations mutually exclusive with *FGFR3* and *KDM6A*, while *TP53* and *RB1* mutations co-occurred, indicating potential mutual exclusive and cooperative roles in tumorigenesis, respectively (54, 55). In addition, *TP53* and *TERT* mutations were enriched in metastatic tumours, whereas *RB1*, *KDM6A*, and *KMT2D* were more common in primary tumours. No significant differences were observed for *ARID1A*, *FGFR3*, or *CDKN2A* between metastatic and primary tumours in such cohort.

These divergent mutation patterns support the existence of specific tumour molecular subtypes. Previous studies showed that bladder cancers harbouring concurrent *RB1* and *TP53* alterations consistently exhibit multiple features linked to better immunotherapy response, including stronger immune infiltration, enhanced interferon signalling, and reduced TGF-β activity (56). They also exhibit higher proliferation, higher TMB and increased tumour-infiltrating lymphocytes. Together, these characteristics suggest that dual *RB1/TP53* loss defines a more immunogenic bladder cancer subtype with elevated sensitivity to immune-checkpoint inhibition.

The identification of *TERT*p and *TP53* mutations in urothelial bladder carcinoma has been reported to have important clinical implications (57). TERTp mutations have been shown to consistently predict improved immunotherapy outcomes independently from the TP53 status, suggesting that enhanced *TERT* activity may increase responsiveness to PD-1/PD-L1 blockade. In addition, TERTp mutations or TERT overexpression have been described to define a biologically aggressive, stage-specific subset of urothelial carcinoma, linked to poor survival and platinum resistance in advanced disease but better responses to PD-1/PD-L1 blockade, highlighting the potential of telomerase activation as context-specific biomarker (57).

On the other hand, patients with TP53-mutant bladder carcinoma might benefit from emerging therapeutic approaches aimed at restoring wild-type p53 function, such as small molecules, gene therapy and other strategies (45). Similarly, patients with *TERT*p-mutant tumours may be candidates for therapeutic approaches that directly inhibit telomerase or target signalling pathways linked to telomerase activation (58). Integrating *TERT*p/*TP53* mutation status into clinical decision-making could refine prognostic models and guide the development of combination therapies that target telomerase and/or mutant TP53 in conjunction with immunotherapy approaches.

The association of *TERT*p and *TP53* mutations has been studied in other types of cancer such as hepatocellular carcinoma, brain cancer and melanoma (59, 60). In hepatocellular carcinoma, *TERT*p and *TP53* co-mutations were more frequently identified in male patients, in moderately differentiated tumours and associated with a significant higher risk of tumour relapse and shorter progression-free survival (59). The frequent coexistence of *TERT*p and *TP53* mutations in bladder cancer with high tumour grade and poor outcomes supports their association with an aggressive tumour phenotype. However, it remains unclear whether these mutations directly drive tumour aggressiveness or they represent markers of later disease stages.

In our analyses, TERTp and TP53 mutation status in bladder cancer patients who did not received intravesical or systemic therapy was significantly associated with poorer overall survival in univariate analyses and retained independent prognostic significance in the multivariate model when adjusted for age, smoking status, and tumour grade. These results suggests that *TERT*p and *TP53* mutations have a robust, independent association with adverse outcomes and supports their role as biologically distinct cancer drivers in urothelial carcinoma.

Our study has several limitations. First, its retrospective design and reliance on publicly available datasets may introduce selection bias. Second, while our analysis provides detailed analysis on the associations between *TERT*p and *TP53* mutations, experimental validation of their cooperative role is needed to clarify the underlying biological mechanisms. Third, we attempted to identify independent validation cohorts with comprehensive *TERTp* and *TP53* mutation data linked to clinical outcomes, however, publicly available datasets covering non-coding regions of human cancer genomes are currently limited, including urothelial carcinoma. Finally, our study does not address potential therapeutic implications, and prospective studies integrating mechanistic experiments and treatment response data are required to confirm these associations and evaluate the feasibility of targeting *TERT*p and/or *TP53* co-mutations in clinical interventions. Nevertheless, the findings might provide valuable clinical observations to guide future research to identify predictive biomarkers of clinical outcome and therapeutic response.

## Conclusions

Further research, including large-scale genomic analyses and prospective clinical studies, is needed to better understand the interplay between *TERT*p mutations, variations in cancer driver genes, environmental risk factors, and clinical outcomes in bladder cancer. Clarifying these relationships could potentially lead to the development of more personalized prognostic markers and therapeutic strategies for this disease.

## Data Availability

The data used in this study were obtained from the open-access source cBioPortal (https://www.cbioportal.org, 21 January 2026). Datasets included in the analyses are described in the Supplementary Materials. Additional information, including analysis scripts, is available from the corresponding author upon reasonable request.

## References

[1] Bray F Laversanne M Sung H Ferlay J Siegel RL Soerjomataram I . Global cancer statistics 2022: GLOBOCAN estimates of incidence and mortality worldwide for 36 cancers in 185 countries. CA Cancer J Clin. (2024) 74:229–63. doi: 10.3322/caac.21834, PMID: 38572751

[2] Halaseh SA Halaseh S Alali Y Ashour ME Alharayzah MJ . A review of the etiology and epidemiology of bladder cancer: all you need to know. Cureus. (2022) 14:e27330. doi: 10.7759/cureus.27330, PMID: 36042998 PMC9411696

[3] Freedman ND Silverman DT Hollenbeck AR Schatzkin A Abnet CC . Association between smoking and risk of bladder cancer among men and women. JAMA. (2011) 306:737–45. doi: 10.1001/jama.2011.1142, PMID: 21846855 PMC3441175

[4] van Osch FH Jochems SH van Schooten FJ Bryan RT Zeegers MP . Quantified relations between exposure to tobacco smoking and bladder cancer risk: a meta-analysis of 89 observational studies. Int J Epidemiol. (2016) 45:857–70. doi: 10.1093/ije/dyw044, PMID: 27097748

[5] Cumberbatch MG Cox A Teare D Catto JW . Contemporary occupational carcinogen exposure and bladder cancer: A systematic review and meta-analysis. JAMA Oncol. (2015) 1:1282–90. doi: 10.1001/jamaoncol.2015.3209, PMID: 26448641

[6] Zhang Y . Understanding the gender disparity in bladder cancer risk: the impact of sex hormones and liver on bladder susceptibility to carcinogens. J Environ Sci Health C Environ Carcinog Ecotoxicol Rev. (2013) 31:287–304. doi: 10.1080/10590501.2013.844755, PMID: 24171436 PMC3852434

[7] Sanli O Dobruch J Knowles MA Burger M Alemozaffar M Nielsen ME . Bladder cancer. Nat Rev Dis Primers. (2017) 3:17022. doi: 10.1038/nrdp.2017.22, PMID: 28406148

[8] Burger M Catto JW Dalbagni G Grossman HB Herr H Karakiewicz P . Epidemiology and risk factors of urothelial bladder cancer. Eur Urol. (2013) 63:234–41. doi: 10.1016/j.eururo.2012.07.033, PMID: 22877502

[9] Kaufman DS Shipley WU Feldman AS . Bladder cancer. Lancet. (2009) 374:239–49. doi: 10.1016/S0140-6736(09)60491-8, PMID: 19520422

[10] Epstein JI Amin MB Reuter VR Mostofi FK . The World Health Organization/International Society of Urological Pathology consensus classification of urothelial (transitional cell) neoplasms of the urinary bladder. Bladder Consensus Conference Committee. Am J Surg Pathol. (1998) 22:1435–48. doi: 10.1097/00000478-199812000-00001, PMID: 9850170

[11] Humphrey PA Moch H Cubilla AL Ulbright TM Reuter VE . The 2016 WHO classification of tumours of the urinary system and male genital organs-part B: prostate and bladder tumours. Eur Urol. (2016) 70:106–19. doi: 10.1016/j.eururo.2016.02.028, PMID: 26996659

[12] Boustead GB Fowler S Swamy R Kocklebergh R Hounsome L . Section of Oncology B. Stage, grade and pathological characteristics of bladder cancer in the UK: British Association of Urological Surgeons (BAUS) urological tumour registry. BJU Int. (2014) 113:924–30. doi: 10.1111/bju.12468, PMID: 24447825

[13] Fernandez-Gomez J Madero R Solsona E Unda M Martinez-Pineiro L Gonzalez M . Predicting nonmuscle invasive bladder cancer recurrence and progression in patients treated with bacillus Calmette-Guerin: the CUETO scoring model. J Urol. (2009) 182:2195–203. doi: 10.1016/j.juro.2009.07.016, PMID: 19758621

[14] Sylvester RJ van der Meijden AP Oosterlinck W Witjes JA Bouffioux C Denis L . Predicting recurrence and progression in individual patients with stage Ta T1 bladder cancer using EORTC risk tables: a combined analysis of 2596 patients from seven EORTC trials. Eur Urol. (2006) 49:466–5. doi: 10.1016/j.eururo.2005.12.031, PMID: 16442208

[15] Ravvaz K Walz ME Weissert JA Downs TM . Predicting nonmuscle invasive bladder cancer recurrence and progression in a United States population. J Urol. (2017) 198:824–31. doi: 10.1016/j.juro.2017.04.077, PMID: 28433642

[16] Prout GR Marshall VF . The prognosis with untreated bladder tumors. Cancer. (1956) 9:551–8. doi: 10.1002/1097-0142(195605/06)9:3<551::aid-cncr2820090319>3.0.co;2-2 13330006

[17] van Doeveren T van de Werken HJG van Riet J Aben KKH van Leeuwen PJ Zwarthoff EC . Synchronous and metachronous urothelial carcinoma of the upper urinary tract and the bladder: Are they clonally related? A systematic review. Urol Oncol. (2020) 38:590–8. doi: 10.1016/j.urolonc.2020.01.008, PMID: 32057596

[18] Nordentoft I Lamy P Birkenkamp-Demtroder K Shumansky K Vang S Hornshoj H . Mutational context and diverse clonal development in early and late bladder cancer. Cell Rep. (2014) 7:1649–63. doi: 10.1016/j.celrep.2014.04.038, PMID: 24835989

[19] Lamy P Nordentoft I Birkenkamp-Demtroder K Thomsen MB Villesen P Vang S . Paired exome analysis reveals clonal evolution and potential therapeutic targets in urothelial carcinoma. Cancer Res. (2016) 76:5894–906. doi: 10.1158/0008-5472.CAN-16-0436, PMID: 27488526

[20] Thomsen MBH Nordentoft I Lamy P Vang S Reinert L Mapendano CK . Comprehensive multiregional analysis of molecular heterogeneity in bladder cancer. Sci Rep. (2017) 7:11702. doi: 10.1038/s41598-017-11291-0, PMID: 28916750 PMC5600970

[21] Alexandrov LB Nik-Zainal S Wedge DC Aparicio SA Behjati S Biankin AV . Signatures of mutational processes in human cancer. Nature. (2013) 500:415–21. doi: 10.1038/nature12477, PMID: 23945592 PMC3776390

[22] Lawrence MS Stojanov P Polak P Kryukov GV Cibulskis K Sivachenko A . Mutational heterogeneity in cancer and the search for new cancer-associated genes. Nature. (2013) 499:214–8. doi: 10.1038/nature12213, PMID: 23770567 PMC3919509

[23] Robertson AG Kim J Al-Ahmadie H Bellmunt J Guo G Cherniack AD . Comprehensive molecular characterization of muscle-invasive bladder cancer. Cell. (2018) 174:1033. doi: 10.1016/j.cell.2018.07.036, PMID: 30096301 PMC6297116

[24] Yuen K Meagher M Mercer J Yilma B Stoppler M Fragkogianni S . Comprehensive comparison of somatic, germline, and immune cell profiles in upper tract and bladder urothelial carcinoma. JCO Precis Oncol. (2025) 9:e2500289. doi: 10.1200/PO-25-00289, PMID: 40773709

[25] Spruck CH 3rd Rideout WM 3rd Olumi AF Ohneseit PF Yang AS Tsai YC . Distinct pattern of p53 mutations in bladder cancer: relationship to tobacco usage. Cancer Res. (1993) 53:1162–6. 8439962

[26] Sidransky D Von Eschenbach A Tsai YC Jones P Summerhayes I Marshall F . Identification of p53 gene mutations in bladder cancers and urine samples. Science. (1991) 252:706–9. doi: 10.1126/science.2024123, PMID: 2024123

[27] Fujimoto K Yamada Y Okajima E Kakizoe T Sasaki H Sugimura T . Frequent association of p53 gene mutation in invasive bladder cancer. Cancer Res. (1992) 52:1393–8. 1540947

[28] Shay JW Wright WE . Hayflick, his limit, and cellular ageing. Nat Rev Mol Cell Biol. (2000) 1:72–6. doi: 10.1038/35036093, PMID: 11413492

[29] Yuan X Larsson C Xu D . Mechanisms underlying the activation of TERT transcription and telomerase activity in human cancer: old actors and new players. Oncogene. (2019) 38:6172–83. doi: 10.1038/s41388-019-0872-9, PMID: 31285550 PMC6756069

[30] Horn S Figl A Rachakonda PS Fischer C Sucker A Gast A . TERT promoter mutations in familial and sporadic melanoma. Science. (2013) 339:959–61. doi: 10.1126/science.1230062, PMID: 23348503

[31] Huang FW Hodis E Xu MJ Kryukov GV Chin L Garraway LA . Highly recurrent TERT promoter mutations in human melanoma. Science. (2013) 339:957–9. doi: 10.1126/science.1229259, PMID: 23348506 PMC4423787

[32] Gupta S Vanderbilt CM Lin YT Benhamida JK Jungbluth AA Rana S . A pan-cancer study of somatic TERT promoter mutations and amplification in 30,773 tumors profiled by clinical genomic sequencing. J Mol Diagn. (2021) 23:253–63. doi: 10.1016/j.jmoldx.2020.11.003, PMID: 33285287 PMC7874333

[33] Heidenreich B Kumar R . Altered TERT promoter and other genomic regulatory elements: occurrence and impact. Int J Cancer. (2017) 141:867–76. doi: 10.1002/ijc.30735, PMID: 28407294

[34] Allory Y Beukers W Sagrera A Flandez M Marques M Marquez M . Telomerase reverse transcriptase promoter mutations in bladder cancer: high frequency across stages, detection in urine, and lack of association with outcome. Eur Urol. (2014) 65:360–6. doi: 10.1016/j.eururo.2013.08.052, PMID: 24018021

[35] Arita H Narita Y Takami H Fukushima S Matsushita Y Yoshida A . TERT promoter mutations rather than methylation are the main mechanism for TERT upregulation in adult gliomas. Acta Neuropathol. (2013) 126:939–41. doi: 10.1007/s00401-013-1203-9, PMID: 24174165

[36] Sizemore GM Pitarresi JR Balakrishnan S Ostrowski MC . The ETS family of oncogenic transcription factors in solid tumours. Nat Rev Cancer. (2017) 17:337–51. doi: 10.1038/nrc.2017.20, PMID: 28450705

[37] Tornesello ML Cerasuolo A Starita N Amiranda S Bonelli P Tuccillo FM . Reactivation of telomerase reverse transcriptase expression in cancer: the role of TERT promoter mutations. Front Cell Dev Biol. (2023) 11:1286683. doi: 10.3389/fcell.2023.1286683, PMID: 38033865 PMC10684755

[38] Agarwal N Rinaldetti S Cheikh BB Zhou Q Hass EP Jones RT . TRIM28 is a transcriptional activator of the mutant TERT promoter in human bladder cancer. Proc Natl Acad Sci U.S.A. (2021) 118:e2102423118. doi: 10.1073/pnas.2102423118, PMID: 34518220 PMC8463889

[39] Hurst CD Platt FM Knowles MA . Comprehensive mutation analysis of the TERT promoter in bladder cancer and detection of mutations in voided urine. Eur Urol. (2014) 65:367–9. doi: 10.1016/j.eururo.2013.08.057, PMID: 24035680

[40] Liu X Wu G Shan Y Hartmann C von Deimling A Xing M . Highly prevalent TERT promoter mutations in bladder cancer and glioblastoma. Cell Cycle. (2013) 12:1637–8. doi: 10.4161/cc.24662, PMID: 23603989 PMC3680543

[41] Zehir A Benayed R Shah RH Syed A Middha S Kim HR . Mutational landscape of metastatic cancer revealed from prospective clinical sequencing of 10,000 patients. Nat Med. (2017) 23:703–13. doi: 10.1038/nm.4333, PMID: 28481359 PMC5461196

[42] Hosen MI Sheikh M Zvereva M Scelo G Forey N Durand G . Urinary TERT promoter mutations are detectable up to 10 years prior to clinical diagnosis of bladder cancer: Evidence from the Golestan Cohort Study. EBioMedicine. (2020) 53:102643. doi: 10.1016/j.ebiom.2020.102643, PMID: 32081602 PMC7118568

[43] Liu Y Su Z Tavana O Gu W . Understanding the complexity of p53 in a new era of tumor suppression. Cancer Cell. (2024) 42:946–67. doi: 10.1016/j.ccell.2024.04.009, PMID: 38729160 PMC11190820

[44] Levine AJ . p53: 800 million years of evolution and 40 years of discovery. Nat Rev Cancer. (2020) 20:471–80. doi: 10.1038/s41568-020-0262-1, PMID: 32404993

[45] Tornesello ML . TP53 mutations in cancer: Molecular features and therapeutic opportunities (Review). Int J Mol Med. (2025) 557. doi: 10.3892/ijmm.2024.5448, PMID: 39450536 PMC11554381

[46] Barr AR Burley A Wilkins A . TP53 mutations in urothelial carcinoma: not all one and the same(dagger). J Pathol. (2024) 264:125–8. doi: 10.1002/path.6335, PMID: 39046056

[47] Clinton TN Chen Z Wise H Lenis AT Chavan S Donoghue MTA . Genomic heterogeneity as a barrier to precision oncology in urothelial cancer. Cell Rep. (2022) 41:111859. doi: 10.1016/j.celrep.2022.111859, PMID: 36543146 PMC9882421

[48] Iyer G Tangen CM Sarfaty M Regazzi AM Lee IL Fong M . DNA damage response alterations predict for neoadjuvant chemotherapy sensitivity in muscle-invasive bladder cancer: A correlative analysis of the SWOG S1314 trial. JCO Precis Oncol. (2024) 8:e2400287. doi: 10.1200/PO.24.00287, PMID: 39499893 PMC12088707

[49] Piraino SW Furney SJ . Beyond the exome: the role of non-coding somatic mutations in cancer. Ann Oncol. (2016) 27:240–8. doi: 10.1093/annonc/mdv561, PMID: 26598542

[50] Elliott K Larsson E . Non-coding driver mutations in human cancer. Nat Rev Cancer. (2021) 21:500–9. doi: 10.1038/s41568-021-00371-z, PMID: 34230647

[51] Chaudhary P Singha B Abdel-Hafiz HA Velegraki M Sundi D Satturwar S . Sex differences in bladder cancer: understanding biological and clinical implications. Biol Sex Differ. (2025) 16:31. doi: 10.1186/s13293-025-00715-6, PMID: 40361239 PMC12070554

[52] Gunes C Wezel F Southgate J Bolenz C . Implications of TERT promoter mutations and telomerase activity in urothelial carcinogenesis. Nat Rev Urol. (2018) 15:386–93. doi: 10.1038/s41585-018-0001-5, PMID: 29599449

[53] Calvet F Blanco Martinez-Illescas R Muinos F Tretiakova M Latorre-Esteves ES Fredrickson J . Sex and smoking bias in the selection of somatic mutations in human bladder. Nature. (2025) 647:436–44. doi: 10.1038/s41586-025-09521-x, PMID: 41062697 PMC12611770

[54] Braun JP Palattao KAD Jr. Torbenson E Hsia B Tauseef A . Genomic characteristics of bladder cancer: an AACR project GENIE study. Int J Mol Sci. (2025) 26:11653. doi: 10.3390/ijms262311653, PMID: 41373802 PMC12692009

[55] Consortium APG . AACR project GENIE: powering precision medicine through an international consortium. Cancer Discov. (2017) 7:818–31. doi: 10.1158/2159-8290.CD-17-0151, PMID: 28572459 PMC5611790

[56] Manzano RG Catalan-Latorre A Brugarolas A . RB1 and TP53 co-mutations correlate strongly with genomic biomarkers of response to immunity checkpoint inhibitors in urothelial bladder cancer. BMC Cancer. (2021) 21:432. doi: 10.1186/s12885-021-08078-y, PMID: 33879103 PMC8056512

[57] Jin K Xu J Zhang L Liu Z Su X Xu Z . TERT promoter mutations or protein overexpression define an aggressive subset with favourable immunotherapeutic response in advanced urothelial carcinoma. BMJ Oncol. (2025) 4:e000586. doi: 10.1136/bmjonc-2024-000586, PMID: 40099003 PMC11911668

[58] Tornesello ML Tornesello AL Starita N Cerasuolo A Izzo F Buonaguro L . Telomerase: a good target in hepatocellular carcinoma? An overview of relevant preclinical data. Expert Opin Ther Targets. (2022) 26:767–80. doi: 10.1080/14728222.2022.2147062, PMID: 36369706

[59] Li J Bai L Xin Z Song J Chen H Song X . TERT-TP53 mutations: a novel biomarker pair for hepatocellular carcinoma recurrence and prognosis. Sci Rep. (2025) 15:3620. doi: 10.1038/s41598-025-87545-z, PMID: 39880909 PMC11779956

[60] Schwaederle M Krishnamurthy N Daniels GA Piccioni DE Kesari S Fanta PT . Telomerase reverse transcriptase promoter alterations across cancer types as detected by next-generation sequencing: A clinical and molecular analysis of 423 patients. Cancer. (2018) 124:1288–96. doi: 10.1002/cncr.31175, PMID: 29211306 PMC5839978

