# Supplementary material for: Effect of co-occurring mutations in TP53 gene and TERT promoter on the survival of bladder cancer patients
Source: Front Immunol. 2026 Mar 9;17:1771897. doi: 10.3389/fimmu.2026.1771897 (PMC13006593; doi:10.3389/fimmu.2026.1771897)
Supplement: Supplementary file 1 [file DataSheet1.pdf]

Supplementary materials

Effect of co-occurring mutations in *TP53* gene and *TERT* promoter on the survival of bladder cancer patients

Maria Lina Tornesello[1,], Maria Carmela Piccirillo[2], Rosa Tambaro[3], Vittorio Simeon[4]*

[1]Molecular Biology and Viral Oncology Unit, Department of Translational Research, Istituto Nazionale Tumori IRCCS Fondazione G. Pascale, Napoli, Italy

[2]Clinical Trials Unit, Department of Translational Research, Istituto Nazionale Tumori IRCCS Fondazione G. Pascale, Napoli, Italy

[3]Uro-Gynaecological Clinical Experimental Medical Oncology, Department of Urology and Gynecology, Istituto Nazionale Tumori IRCCS Fondazione G. Pascale, Napoli, Italy

[4]Dipartimento di Salute Mentale e Fisica e Medicina Preventiva, Università della Campania L. Vanvitelli.

Table 1S. Summary of publicly available bladder cancer studies accessed via cBioPortal. For each study, the availability of TERTp mutation data and survival time (Surv) is indicated (Yes/No), along with the total number of tumour samples and links to the respective cBioPortal study section.

	Study Title	TERTp	Surv	N. Samples
1.	Bladder Urothelial Carcinoma (TCGA, Firehose Legacy) https://www.cbioportal.org/study/summary?id=blca_tcga	No	Yes	826
2.	Bladder Urothelial Carcinoma (TCGA, GDC) https://www.cbioportal.org/study/summary?id=blca_tcga_gdc	No	Yes	413
3.	Bladder Urothelial Carcinoma (TCGA, PanCancer Atlas) https://www.cbioportal.org/study/summary?id=blca_tcga_pan_can_atlas_2018	No	Yes	411
4.	Bladder (MSK, J Clin Onco 2013) https://www.cbioportal.org/study/summary?id=blca_mskcc_solit_2012	No	No	121
5.	Bladder Urothelial Carcinoma (BGI, Nat Genet 2013 https://www.cbioportal.org/study/summary?id=blca_bgi	No	No	99
6.	Bladder Cancer (MSK, Eur Urol 2014) https://www.cbioportal.org/study/summary?id=blca_mskcc_solit_2014	No	No	109
7.	Bladder Urothelial Carcinoma (DFCI/MSK, Cancer Discov 2014) https://www.cbioportal.org/study/summary?id=blca_dfarber_mskcc_2014	No	No	50
8.	Bladder Urothelial Carcinoma (TCGA, Nature 2014) https://www.cbioportal.org/study/summary?id=blca_mskcc_solit_2012	No	No	262
9.	Nonmuscle Invasive Bladder Cancer (MSK Eur Urol 2017) https://www.cbioportal.org/study/summary?id=blca_nmibc_2017	No	No	105
10.	Bladder Cancer (TCGA, Cell 2017) https://www.cbioportal.org/study/summary?id=blca_tcga_pub_2017	No	Yes	826
11.	Bladder Cancer (Columbia University/MSK, Cell 2018) https://www.cbioportal.org/study/summary?id=bladder_columbia_msk_2018	No	No	130
12.	Urothelial Carcinoma (BCAN/HCRN, Nat Commun 2022) https://www.cbioportal.org/study/summary?id=blca_bcan_hcrn_2022	No	Yes	192
13.	Urothelial Carcinoma (Cornell/Trento, Nat Gen 2016) https://www.cbioportal.org/study/summary?id=blca_cornell_2016	No	No	72
14.	Metastatic Bladder Urothelial Carcinoma (IMvigor210 Phase II Trial, ESMO Open. 2024) - iAtlas Harmonized https://www.cbioportal.org/study/summary?id=blca_iatlas_imvigor210_2017	No	No	347
15.	Bladder Cancer (MSK, Nat Genet 2016) https://www.cbioportal.org/study/summary?id=blca_plasmacytoid_mskcc_2016	Yes	No	34
16.	Bladder Cancer (MSK/TCGA, 2020) https://www.cbioportal.org/study/summary?id=blca_msk_tcga_2020	Partially	Yes	476*
17.	Bladder Cancer (MSK, Cell Reports 2022) https://www.cbioportal.org/study/summary?id=paired_bladder_2022	Yes	Yes	1659
18.	Bladder Cancer (MSK, Clin Cancer Res 2023) https://www.cbioportal.org/study/summary?id=bladder_msk_2023	Yes	Yes	526**
19.	Bladder Cancer (MSK, Eur Urol 2024) https://www.cbioportal.org/study/summary?id=bladder_msk_2024	Yes	No	112***
20.	Bladder Urothelial Carcinoma SWOG S1314 Trial (MSK, JCO Precis Oncol 2024) https://www.cbioportal.org/study/summary?id=blca_msk_2024	Yes	No	184
21.	Bladder Cancer (MSK, Eur Urol Open Sci 2025) https://www.cbioportal.org/study/summary?id=blca_msk_2025	Yes	No	308***

* Cases in this study, including MIBC and metastasis, were selected from the MSK Cell Reports (2022).

** Cases in this study, harbouring FGFR2/3 alterations predictive of response to Erdafitinib, were selected from the MSK Cell Reports (2022).

*** Cases in these studies are diagnosed with upper tract urothelial carcinoma

Table 2S. Distribution of mutant genes in *TERT*p wild type versus *TERT*p mutant cancer cases

Gene name	Gene type	TERT promoter Wild type N = 308 (%)	Mutant N = 803	p-value[1]	q-value[2]
CDKN1A	Oncosuppressor	15 (4.9%)	124 (15%)	<0.001	<0.001
RB1	Oncosuppressor	35 (11%)	194 (24%)	<0.001	<0.001
ERBB2	Oncogene	39 (13%)	170 (21%)	0.001	0.008
ARID1A	Oncosuppressor	70 (23%)	258 (32%)	0.002	0.011
PIK3CA	Oncogene	44 (14%)	178 (22%)	0.003	0.013
TSC1	Oncosuppressor	21 (6.8%)	100 (12%)	0.007	0.022
TP53	Oncosuppressor	132 (43%)	416 (52%)	0.008	0.022
ELF3	Oncosuppressor	23 (7.5%)	103 (13%)	0.012	0.029
KMT2A	Oncogene	25 (8.1%)	107 (13%)	0.016	0.036
ERBB3	Oncogene	26 (8.4%)	104 (13%)	0.036	0.073
KMT2D	Oncogene	94 (31%)	205 (26%)	0.093	0.2
CREBBP	Oncosuppressor	38 (12%)	122 (15%)	0.2	0.4
FGFR3	Oncogene	74 (24%)	218 (27%)	0.3	0.4
ERCC2	Oncogene	36 (12%)	108 (13%)	0.4	0.6
EP300	Oncogene	35 (11%)	103 (13%)	0.5	0.7
ATM	Oncosuppressor	41 (13%)	99 (12%)	0.7	0.8
FAT1	Oncosuppressor	35 (11%)	99 (12%)	0.7	0.8
KMT2C	Oncosuppressor	43 (14%)	116 (14%)	0.8	0.9
KDM6A	Oncosuppressor	102 (33%)	270 (34%)	0.9	0.9
STAG2	Oncogene	41 (13%)	104 (13%)	0.9	0.9

[1] Pearson's Chi-squared test

[2] False discovery rate correction for multiple testing

Table 3S. Distribution of mutant genes in *TP53* wild type versus *TP53* mutant cancer cases

Gene name	Gene type	*TP53* gene Wild type N = 563 (%)	Mutant N = 548[1]	p-value[1]	q-value[2]
FGFR3	Oncosuppressor	229 (41%)	63 (11%)	<0.001	<0.001
RB1	Oncosuppressor	52 (9.2%)	177 (32%)	<0.001	<0.001
CDKN1A	Oncosuppressor	88 (16%)	51 (9.3%)	0.001	0.01
ERBB2	Oncogene	88 (16%)	121 (22%)	0.006	0.03
TERTp	Oncogene	387 (69%)	416 (76%)	0.008	0.03
STAG2	Oncogene	88 (16%)	57 (10%)	0.01	0.032
TSC1	Oncosuppressor	74 (13%)	47 (8.6%)	0.015	0.042
KDM6A	Oncosuppressor	206 (37%)	166 (30%)	0.026	0.065
ELF3	Oncosuppressor	75 (13%)	51 (9.3%)	0.035	0.077
KMT2D	Oncogene	137 (24%)	162 (30%)	0.049	0.1
KMT2A	Oncogene	58 (10%)	74 (14%)	0.1	0.2
ARID1A	Oncosuppressor	154 (27%)	174 (32%)	0.1	0.2
CREBBP	Oncosuppressor	88 (16%)	72 (13%)	0.2	0.3
ERCC2	Oncogene	66 (12%)	78 (14%)	0.2	0.3
PIK3CA	Oncogene	64 (11%)	74 (14%)	0.3	0.4
EP300	Oncogene	120 (21%)	102 (19%)	0.3	0.3
ATM	Oncosuppressor	75 (13%)	65 (12%)	0.5	0.5
ERBB3	Oncogene	63 (11%)	67 (12%)	0.6	0.7
FAT1	Oncosuppressor	70 (12%)	64 (12%)	0.7	0.7
KMT2C	Oncosuppressor	79 (14%)	80 (15%)	0.8	0.8

[1] Pearson's Chi-squared test

[2]False discovery rate correction for multiple testing

Table 4S. Test of proportional-hazards assumption has been performed using Schoenfeld residuals

	Variable	rho	Chi2	df	p
Overall Survival	Age	0.123	2.405978	1	0.121
	Smoker	-0.023	0.000512	1	0.982
	Tumour grade	-0.161	4.093219	1	0.043
	Mutation groups	-0.090	2.286715	3	0.515
	All variables		7.665418	6	0.264

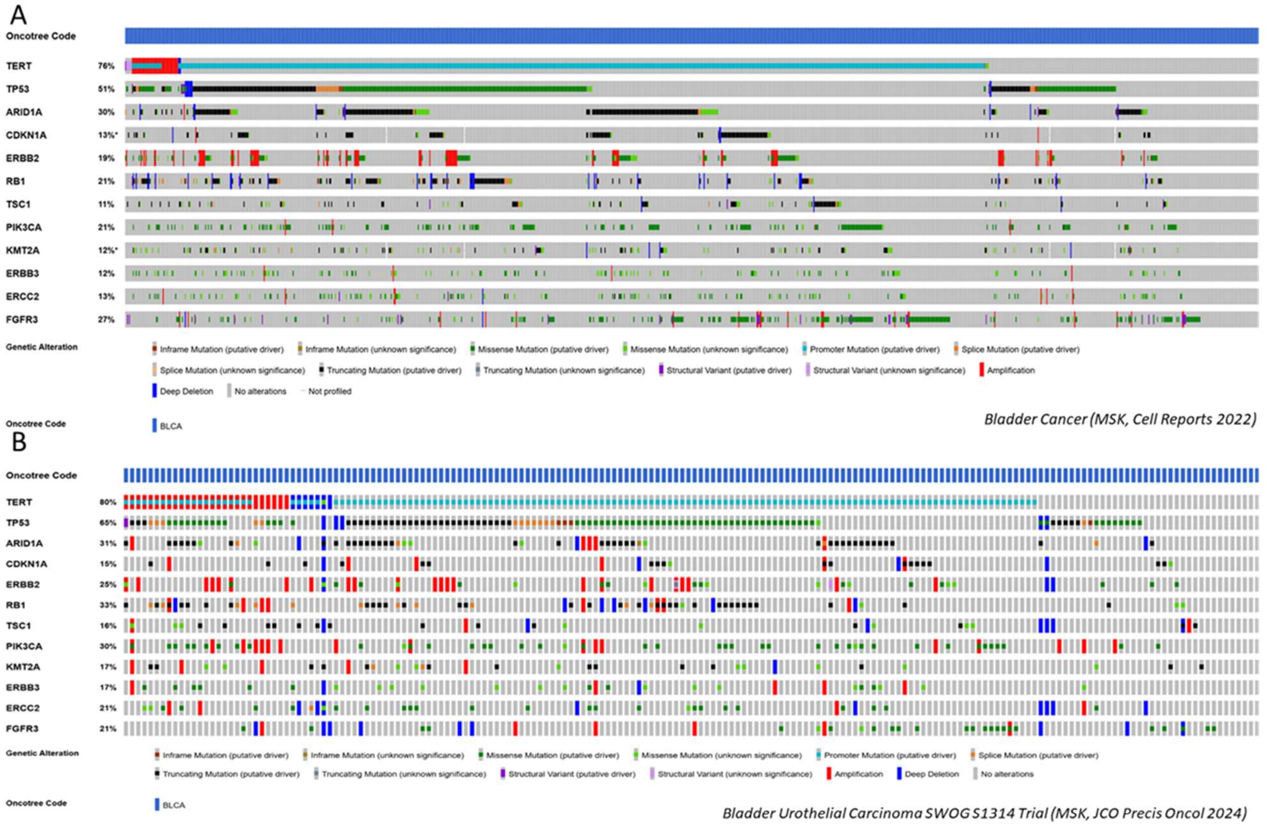

C

wt/wt	TERTp	TP53	TERTp/TP53	p-Value	q-Value	cBioportal study
176	387	132	416	0.008	0.022	*Bladder Cancer (MSK, Cell Reports 2022)*
20	45	18	101	0.021	0.021	*Bladder Urothelial Carcinoma SWOG S1314 Trial (MSK, JCO Precis Oncol 2024)*

Figure 1S. Genomic alterations of *TERT* and *TP53* in bladder cancer. OncoPrint of *TERT* and *TP53* alterations in bladder urothelial carcinoma from the (A) MSK cohort (Cell Reports, 2022) and (B) Bladder Urothelial Carcinoma SWOG S1314 Trial (MSK, JCO Precis Oncol 2024), including somatic mutations in coding and non-coding (notably *TERT*) regions, structural variants, and copy number alterations. Percentages indicate the fraction of tumors with at least one alteration; gray denotes no alteration. (C) Co-occurrence of *TERT*p and *TP53* mutations in the indicated cohort

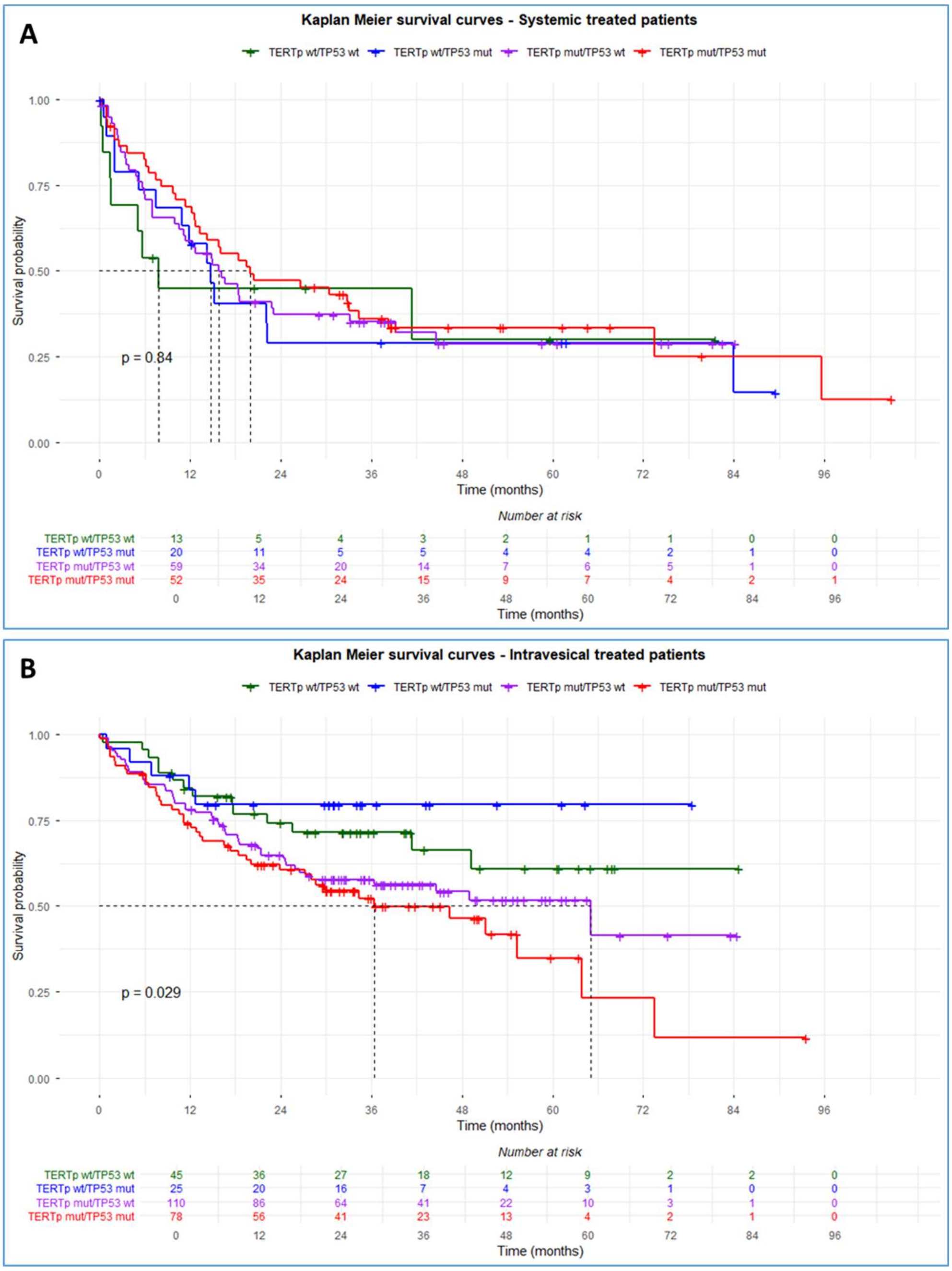

Figure 2S. Kaplan-Meier survival analysis according to *TERT*p and *TP53* mutation status for (A) systemic treated patients and (B) intravesical treated patients.

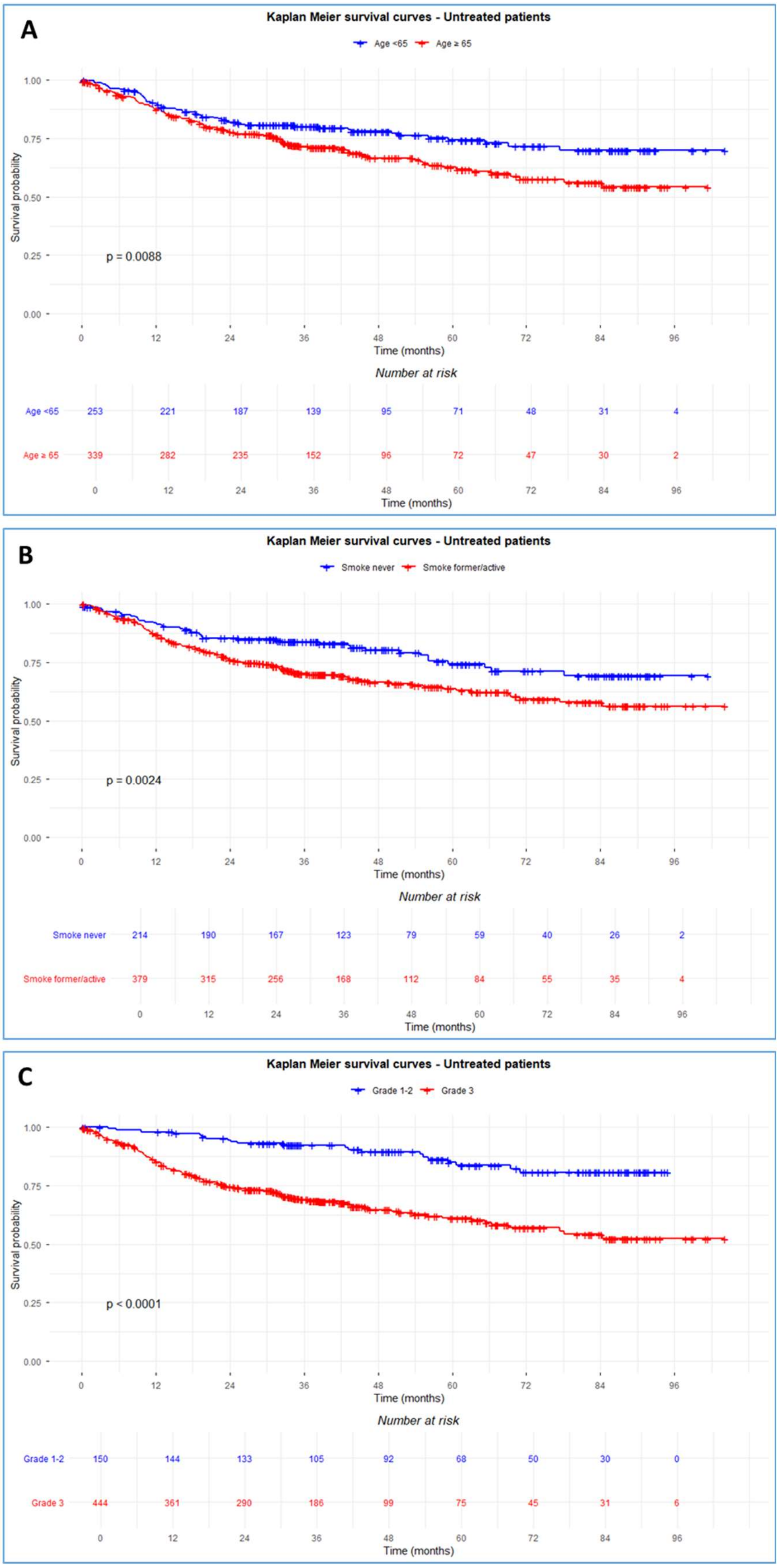

Figure 3S. Kaplan–Meier overall survival curves in untreated patients. Patients' Survival stratified by (A) age (≤65 vs >65 years); (B) by smoking status (never vs former/active), and (C) by tumour grade (Grade 1-2 vs Grade 3). Older age, higher tumour grade, and smoking were each associated with significantly worse overall survival (log-rank p values shown in Table 3).
